# The 24-Hour Window: How Time to Admission Reshapes the Impact of Surgical Delay on Mortality in Elderly Hip Fracture

**DOI:** 10.3390/jcm15145685

**Published:** 2026-07-20

**Authors:** Wei-Song Zhang, Xiao-Long Shao, Zhi Yang, Bin-Fei Zhang

**Affiliations:** Department of Joint Surgery, Honghui Hospital, Xi’an Jiaotong University, No. 555 Youyi East Road, Xi’an 710054, China; zhangweisongz@126.com (W.-S.Z.); shaoxiaolong9527@163.com (X.-L.S.); hhyy_yangzhi@163.com (Z.Y.)

**Keywords:** hip fracture, elderly patients, time to admission, admission to operation, mortality

## Abstract

**Objective:** This study investigated the modifying effect of time to admission (TTA) on the relationship between admission to operation time (ATO) and outcomes in elderly patients with hip fractures. **Methods:** This retrospective cohort study included elderly patients with hip fractures treated at the trauma center. We collected data on patient characteristics, comorbidities, TTA, ATO, and all-cause mortality. Statistical analyses were conducted using R and EmpowerStats software. **Results:** Two thousand three hundred and sixty patients were included, with a median follow-up duration of 39.00 (29.05–50.79) months. Patients were stratified into four groups by ATO: ATO ≤ 2 d; 2 < ATO ≤ 4 d; 4 < ATO ≤ 6 d; and ATO > 6 d. The median TTA for each ATO group was as follows: 24 (4–168), 8 (4–48), 10.5 (4–48), and 10 (4–48) h, respectively. During follow-up, 733 patients (31.07%) died. Interaction analyses further stratified patients by TTA: ≤24 h vs. >24 h. The results demonstrated that TTA exerted a significant modifying effect on the ATO–mortality association. In the TTA ≤ 24 h group, each 1 d ATO delay was associated with a 5% increased risk of mortality (HR = 1.05; 95% CI: 1.01–1.09, *p* = 0.0095). In contrast, ATO delay was not associated with higher mortality in the TTA > 24 h group (HR = 0.98; 95% CI: 0.93–1.02, *p* = 0.3050). Notably, the TTA–ATO–mortality interaction remained statistically significant across several high-risk subgroups: females, those with femoral neck fractures, age-adjusted Charlson comorbidity index ≤ 4, hypertension, coronary artery disease, arrhythmia, and absence of chronic obstructive pulmonary disease. **Conclusions:** TTA is an important effect modifier of the association between ATO and mortality among elderly patients with hip fractures.

## 1. Introduction

High disability rates, high mortality, and a substantial socioeconomic burden characterize elderly hip fractures. Previous research indicates that only 31% of elderly patients regain their pre-fracture ability to perform daily activities one year after hip fracture surgery [1]. Optimizing the clinical management process for patients with hip fractures, especially the timing of surgery, has become crucial in improving the prognosis.

Currently, international consensus emphasizes the importance of the time from admission to operation (ATO). Several guidelines recommend completing the surgery within 24–48 h of the patient’s admission [2,3,4,5]. Early surgery can significantly reduce the risk of postoperative complications, shorten the length of hospital stay, and improve functional recovery. However, ATO delays remain common in clinical practice. Notably, “early surgery” definitions vary across studies, often using different time thresholds. For example, some studies use 48 h as the cutoff for early surgery, and any delay beyond 48 h is considered an ATO delay. A study in the United States reported that 83.7% of patients underwent surgery within 48 h of admission [6], 82.6% in Canada [7], and 43% of elderly hip fracture patients in Spain [8]. In a retrospective study from China, only 39.51% of patients underwent surgery within 48 h of admission [9]. Other studies use a 24 h cutoff for early surgery. Pincus et al. found that patients who underwent hip fracture surgery within 24 h of hospital arrival had lower 30-day mortality and fewer complications, suggesting that an ATO exceeding 24 h might serve as a threshold for increased risk [10]. Surgery rates within 24 h have been reported as 23.5% in the United States [11] and 74.3% in Japan [12]. Additional definitions of early surgery exist, with thresholds of 12 h [13] and 72 h [14]. Such inconsistent cutoff thresholds for early surgery may partially explain the heterogeneous conclusions across studies investigating the association between ATO and mortality after hip fracture. A recent randomized controlled trial found that accelerated surgery did not significantly reduce mortality risk or the incidence of major complications compared to standard care [15]. These inconsistencies highlight the need for further research into the association between ATO and mortality in hip fracture patients.

Time to admission (TTA), from injury to admission, is another important factor associated with prognosis. Delays in TTA can postpone the start of admission assessments and delay perioperative care for elderly patients. Conversely, patients who receive prompt medical attention after injury, resulting in a shorter TTA, tend to experience fewer perioperative pathophysiological changes and shorter ATO, which may improve their long-term survival prospects.

Studies have shown that delayed TTA is associated with a 9% lower post-discharge survival rate and a 7% reduction in one-year post-surgical survival [16]. Notably, a TTA delay exceeding one week is associated with a 76% increase in one-year mortality [17]. Multiple pre-hospital determinants independently shape the length of TTA, including emergency medical service efficiency, residential distance to trauma centers, and inter-hospital transfers. Additionally, numerous studies indicate that factors such as ambulance use and residential location are associated with elderly patients’ social support levels. For example, Asano et al. found that among older adults in Japan, those without someone to listen to their concerns were significantly more likely to use ambulance services than their counterparts with social support [18]. Similarly, patients arriving by ambulance have been shown to have lower social support than those using private transport [19]. Furthermore, widely recognized urban–rural disparities in emergency medical services further highlight this connection, as the spatial distribution of emergency stations plays a critical role in service efficiency and equity [20]. In hip fractures, the social support system also impacts patient outcomes: perceived functional support is positively associated with rehabilitation results [20], and patients with pre-existing limitations in daily activities who lack social support face a high risk of persistent or worsening activity restrictions post-surgery [21]. TTA is influenced by multiple pre-hospital factors, including geographic location, emergency medical service efficiency, hospital referral pathways, and patient/family recognition of injury severity.

This study aims to explore how TTA modifies the relationship between ATO and outcomes in elderly patients with hip fractures, offering new insights into optimizing intervention timing for this patient population.

## 2. Patients and Methods

### 2.1. Study Design

In this retrospective cohort study, we screened patients with hip fractures from 1 January 2015 through 30 September 2019, at Honghui Hospital. This study received approval from the Ethics Committee of our hospital (Approval No. 202201009), and the Institutional Review Boards granted a waiver for informed consent from participants. This waiver was granted based on the study’s retrospective nature, the minimal risk to participants, and the anonymized processing of all data. All procedures involving human subjects have adhered to the ethical standards outlined in the 1964 Declaration of Helsinki and its subsequent amendments. The work has been reported in line with the STROCSS 2025 criteria [22,23].

### 2.2. Participants

Demographic and clinical data for eligible patients were collected from their original medical records. The inclusion criteria were as follows: (1) Age ≥ 65 years; (2) X-ray or computed tomography (CT) diagnosis of the femoral neck or intertrochanteric fracture; (3) patients who received surgical treatment in hospitalization; (4) availability of in-hospital clinical data; (5) patients or their families could be contacted by telephone.

The exclusion criteria were as follows: (1) The presence of compound injury. (2) Severe multiple injuries caused by a car accident or falling from a height, with the patient in a critical condition upon admission. (3) TTA (hours) was defined as the time interval from injury onset to hospital admission, and it was recorded in the medical records; ATO (days) was the time of operation to the time of admission. The time was accurate according to the medical records. Patients were excluded if exact timestamps of injury, admission, and surgery were missing or recorded erroneously in medical charts. (4) The phone number on the front page of the patient’s medical record was incorrect and the patient could not be reached, or the patient’s family members changed the phone number, or the wrong phone number was dialed and the person contacted was neither the patient nor a family member. (5) We could not obtain the patient’s endpoint for other reasons.

### 2.3. Hospital Treatment

After the patient is admitted to the department from the emergency room or an outpatient clinic, an electrocardiogram and blood tests are performed first. An ultrasound examination of the heart and lower extremities is performed for patients with stable conditions. If the patient has no obvious contraindications, low-molecular-weight heparin is administered for anticoagulation to prevent deep vein thrombosis of the lower extremities.

Surgical treatment can be arranged once the patient’s preoperative evaluation reveals no notable findings. The treatment of hip fractures is divided into three types based on fracture type, patient age, and activity level: closed/open reduction and internal fixation (CRIF/ORIF), hemiarthroplasty (HA), and total hip arthroplasty (THA). Generally speaking, for patients with intertrochanteric fractures of the femur, closed/open reduction and internal fixation (CRIF/ORIF) are usually adopted; for patients with femoral neck fractures, if the patient has a high activity level, THA is usually carried out, and if the patient is elderly and has a low activity level, HA treatment is selected. The chief and associate chief surgeons perform the surgical procedures.

### 2.4. Follow-Up

We contacted patients’ families by telephone from January 2022 to March 2022 and recorded postoperative survival data. The survival time was calculated from the start of “time of surgery”. Two medical professionals conducted the telephone follow-up after 2 weeks of training and 1 year of experience. Up to two additional telephone follow-up attempts were conducted for patients unreachable at the first call. If the patients’ family members did not respond, the patients were recorded as lost to follow-up. Survival time was calculated as the precise interval from the date of surgery to the date of death or the last telephone follow-up. Through standardized telephone interviews, the two follow-up physicians meticulously inquired concerning the patient and recorded the exact date of death from the families of deceased patients, thereby determining the survival time from post-operation to death or the last follow-up.

### 2.5. Endpoint Events

This study defined mortality as all-cause mortality about 3 years after operation.

### 2.6. Variables

The following variables were collected and analyzed in our study based on our clinical experience and previous reports: age, sex, injury mechanism, fracture classification, age-adjusted Charlson comorbidity index (aCCI), hypertension, diabetes, arrhythmia, coronary artery disease (CHD), hemorrhagic stroke, dementia, ischemic stroke, cancer, associated injuries, chronic obstructive pulmonary disease (COPD), hepatitis, gastritis, TTA, ATO, albumin, treatment strategy, operation time, blood loss, infusion, transfusion, follow up, mortality. The dependent variable was all-cause mortality, and the independent variable was ATO. ATO was defined as the number of days from admission to the operation. ATO was analyzed as both a continuous variable (per 1 d increase) and a categorical variable (four groups: ≤2 d, 2–4 d, 4–6 d, >6 d). TTA was used as a continuous variable, and 24 h was chosen as the cutoff based on our previous study [24], as shown in Supplementary Figure S1. Other variables were categorized as potential confounding factors.

### 2.7. Statistics Analysis

Descriptive statistical analyses were performed using standard reporting methods. Continuous variables were reported as the mean with standard deviation for normally distributed data, or as the median with interquartile range (IQR) for non-normally distributed data. Categorical variables were reported as percentages. Chi-square tests (categorical variables), one-way ANOVA (normal distribution), or the Kruskal–Wallis H test (skewed distribution) were used to detect differences among ATO groups.

Given the study’s aim to explore long-term risk patterns over the disease course, we selected the Cox model, which offers greater statistical power for analyzing time-to-event data. The Cox models used “time of surgery” as the start of survival time. We constructed crude and adjusted univariate and multivariate Cox proportional hazards models to analyze the association between ATO and mortality. For the Cox proportional hazards regression, the proportional hazards assumption was validated using Schoenfeld residuals. As for the selection of confounding factors, we used clinically relevant factors reported in previous literature [24,25,26,27], factors with *p* < 0.1 in univariate analysis, and factors with effect size changes of more than 10% after adding the factor.

We also performed a sensitivity analysis to verify the robustness of the results. We converted ATO into a categorical variable. We calculated the *p*-value for the trend test to confirm the results of ATO as a continuous variable and examine the possibility of nonlinearity.

Subgroup analyses were performed to explore the consistency of the interaction effect across different patient populations. These analyses were exploratory and were not pre-specified. No adjustment for multiple comparisons (e.g., Bonferroni correction) was applied due to their exploratory nature. Therefore, findings from subgroup analyses should be interpreted with caution and considered hypothesis-generating rather than confirmatory.

We performed a pre-specified interaction effect analysis to explore whether TTA modifies the association between ATO and mortality. The interaction term ”ATO-TTA” was included in the Cox proportional hazards model. The interaction analysis was pre-specified based on our previous findings regarding the relationship between TTA and mortality [24,28]. The fully adjusted model included the following covariates: age, sex, aCCI, albumin level, transfusion, and infusion. These covariates were selected based on univariate analysis and clinical relevance from previous literature. Also, we used “time of admission” as time zero to assess robustness of interaction effect used.

To control for confounding by indication, we performed propensity score matching (PSM) to balance baseline covariates. We performed 1:1 nearest-neighbor propensity score matching without replacement, with a caliper set at 0.05 times the standard deviation of the logit propensity score. Covariate balance post-matching was evaluated via standardized mean differences, with a value of *p* < 0.05 considered indicative of good balance.

All analyses were performed using the statistical software package R (http://www.R-project.org, R Foundation, R version 4.5.3) and EmpowerStats (http://www.empowerstats.com, X&Y Solutions Inc., Boston, MA, USA, version 5.0). Hazard ratios (HR) with 95% confidence intervals (CI) were calculated. A *p*-value < 0.05 (two-sided) was considered to represent statistical significance.

## 3. Results

### 3.1. Patient Characteristics

**Figure 1:**
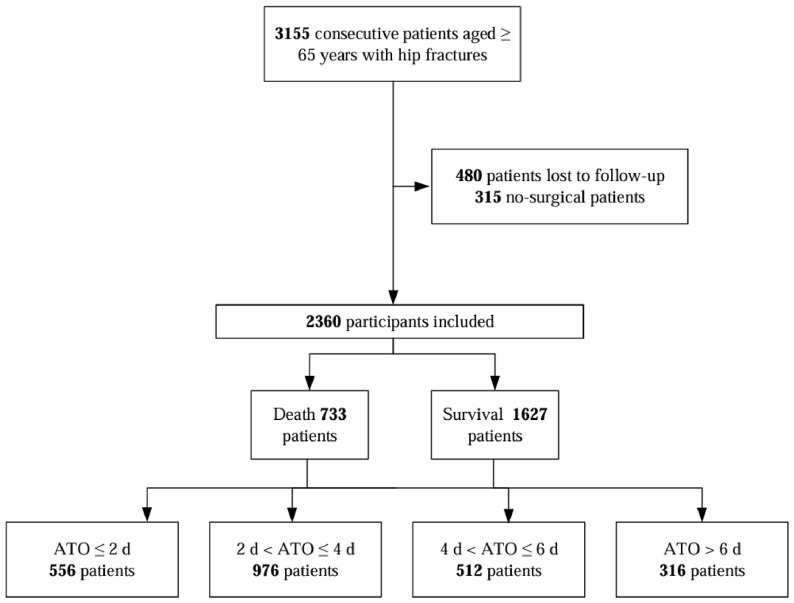
Flow diagram of this study.

A total of 2360 patients were included in the final analysis. The patient selection process is illustrated in Figure 1. Of the 3155 patients initially screened, 795 were excluded for the following reasons: 480 were lost to follow-up and 315 received the non-surgical treatment. Table 1 presents the baseline characteristics of the participants. The median follow-up was 39.00 (29.05–50.79) months. Patients were divided into four groups: ATO ≤ 2 d; 2 d < ATO ≤ 4 d; 4 d < ATO ≤ 6 d; ATO > 6 d. The mean age was 79.47 ± 6.68, 79.54 ± 6.65, 79.13 ± 6.74 and 79.57 ± 6.80 years, respectively. We observed that there were no statistically significant differences among the four groups of patients in terms of age, sex, injury mechanism, fracture classification, hypertension, hemorrhagic stroke, ischemic stroke, cancer, associated injuries, dementia, hepatitis, gastritis, treatment strategy, and blood loss.

At the same time, we observed that patients in group ATO > 6 d had higher aCCI and more comorbidities, such as diabetes, CHD, arrhythmia, and COPD, as well as longer operation time, infusion, blood transfusion, and a higher mortality rate (*p* < 0.05).

Overall median TTA was 10 (IQR: 4–48) h. The median TTA for each ATO group was as follows: 24 (4–168), 8 (4–48), 10.5 (4–48), and 10 (4–48) h, respectively. The median TTA in the ATO ≤ 2 d group was 24 h (IQR: 4–168), reflecting the highly skewed distribution with some patients experiencing very long pre-hospital delays despite prompt in-hospital surgery.

During the follow-up period, a total of 733 (31.07%) patients died (ATO ≤ 2 d: 173 (31.12%); 2 d < ATO ≤ 4 d: 273 (27.97%); 4 d < ATO ≤ 6 d: 170 (33.20%); ATO > 6 d: 117 (37.03%)).

### 3.2. Univariate Analysis of Potential Confounders

To explore the relationship between ATO and mortality during long-term follow-up, we conducted a univariate Cox regression analysis to identify the confounding factors that needed to be adjusted. The dependent variable was the all-cause mortality during long-term follow-up. Potential confounding factors were selected based on previous literature and statistical criteria. Age, sex, aCCI, albumin, and TTA were identified as potential confounders from previous studies [24,25,26,27]. Transfusion and infusion were additionally included because their inclusion resulted in a change in effect size > 10% and met the criterion of *p* < 0.1. Accordingly, age, sex, aCCI, albumin, TTA, transfusion, and infusion were adjusted for in the multivariate analysis (Table 2).

### 3.3. Multivariate Analysis of ATO and Mortality

The univariate analysis did not find a linear association between ATO and mortality during long-term follow-up. To further explore the relationship between them, we used three models to establish an association between ATO and long-term mortality under various data types of ATO. When ATO was treated as a continuous variable, no linear relationship was found between ATO and mortality, whether in the minimally adjusted model (HR = 1.02; 95% CI: 0.99–1.05; *p* = 0.1541) or the fully adjusted model (HR = 1.01; 95% CI: 0.99–1.04; *p* = 0.3134), as shown in Table 3. When ATO was considered as an unordered categorical variable, in the fully adjusted model, we found that compared with the ATO ≤ 2 d group, the risk of mortality in the 2 d < ATO ≤ 4 d group decreased by 12% (HR = 0.88; 95% CI: 0.72–1.09, *p* = 0.2408), decreased 7% (HR = 0.93; 95% CI: 0.73–1.18, *p* = 0.5401) in the 4 d < ATO ≤ 6 d group, and the risk of mortality in the ATO > 6 d group increased by 10% (HR = 1.10; 95% CI: 0.85–1.43, *p* = 0.4512). However, these results did not reach statistical significance.

In addition, we also explored the *p* for trend by treating the three ATO groups as an ordered categorical variable. The *p* for trend in each group was greater than 0.05. Therefore, from the perspective of a linear relationship, there is no correlation between ATO and long-term mortality.

### 3.4. The Interaction Role of TTA on the Association Between ATO and Mortality

In the univariate analysis, we identified several factors associated with ATO. Moreover, some of these factors have been previously reported to be related to the prognosis of elderly patients with hip fractures. Therefore, it is necessary to explore the interactions among different factors. The interaction factors we explored were as follows: sex, aCCI, TTA, hypertension, CHD, arrhythmia, ischemic stroke, and COPD.

Among the sex, comorbidities, and aCCI, we did not detect any interaction. However, the interaction was obvious in TTA, as shown in Table 4.

According to the results of our previous study, TTA was divided into two groups: TTA ≤ 24 h and TTA > 24 h. In the unadjusted model, the interaction *p*-value was 0.0036. After adjusting for age, gender, aCCI, blood transfusion, and infusion volume, 1607 patients were analyzed in the TTA ≤ 24 h group. The ATO delay was associated with a 5% increase in the mortality rate of patients (HR = 1.05; 95% CI: 1.01–1.09, *p* = 0.0095). In the TTA > 24 h group, 681 patients were analyzed, and ATO delay was not associated with an increase in the mortality of patients (HR = 0.98; 95% CI: 0.93–1.02, *p* = 0.3050). The final interaction *p*-value was 0.0109. Therefore, TTA modified the relationship between ATO and patient prognosis, and patients with TTA ≤ 24 h were more sensitive to ATO delay.

Also, we repeated the primary interaction analysis model after sequentially excluding cases with TTA > 168 h (1 week) or >336 h (2 weeks). After excluding TTA > 1 week, the sample size was 2019. The interaction *p*-value was 0.0292. In the TTA ≤ 24 h subgroup, the HR per day ATO delay was 1.05 (95% CI: 1.01–1.09, *p* = 0.0101); in the TTA > 24 h subgroup, it was 0.99 (95% CI: 0.94–1.04, *p* = 0.6919). After excluding TTA > 2 weeks, the sample size was 2220. The interaction *p*-value was 0.0141. In the TTA ≤ 24 h subgroup, the HR was 1.05 (95% CI: 1.01–1.09, *p* = 0.0093); in the TTA > 24 h subgroup, it was 0.98 (95% CI: 0.93–1.03, *p* = 0.3499). The sensitivity analysis clearly demonstrates that the modifying effect of TTA on the ATO–mortality association remained statistically significant and robust after excluding extreme TTA outliers that may not represent typical clinical presentations. The effect direction and magnitude are essentially unchanged. This strongly supports that our core conclusion is not driven by a few anomalous data points.

To address the potential for immortal time bias, we performed a sensitivity analysis using admission time, rather than surgery time, as the time origin for survival calculation. As shown in Supplementary Table S1, the results of this sensitivity analysis were highly consistent with our primary findings. To compare the differences, we retained the HR values to four decimal places. The modifying effect of TTA on the ATO–mortality relationship remained statistically significant (*p* for interaction = 0.0112). In the TTA ≤ 24 h subgroup, each 1 day ATO delay was still significantly associated with a 5% increase in mortality (HR = 1.0513; 95% CI: 1.0115–1.0927, *p* = 0.0111). Conversely, in the TTA > 24 h subgroup, ATO delay was not significantly associated with mortality (HR = 0.9745; 95% CI: 0.9291–1.0221, *p* = 0.2889). The near-identical HR estimates between the two models demonstrate that our conclusions are robust and not driven by the choice of time origin.

**Figure 2:**
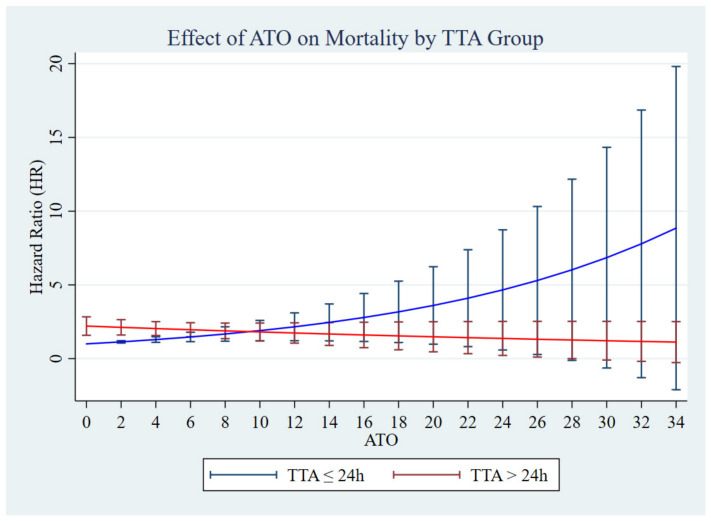
The marginal effects plot for TTA ≤ 24 h and TTA > 24 h. There were 1649 patients in the TTA ≤ 24 h group and 711 in the TTA > 24 h group. The blue line (TTA ≤ 24 h) shows a rising mortality risk with increasing ATO, whereas the red line (TTA > 24 h) shows a stable HR independent of ATO. Vertical bars represent 95% confidence intervals.

Figure 2 shows the marginal effects plot in the TTA ≤ 24 h and TTA > 24 h groups. There was an obvious difference between the TTA ≤ 24 h and TTA > 24 h groups.

### 3.5. PSM

We selected the caliper matching method and set the allowable score difference to 0.05. Finally, 1200 patients (50.8%) were successfully matched after 1:1 matching. Most of the successfully matched patients in the two groups had scores between 0.7 and 0.95, indicating a high level of match. The distribution of propensity scores before and after matching is shown in Supplementary Figure S2, and the covariate balance is presented in Supplementary Table S2. The comparison balance between the two groups after matching is shown in Supplementary Table S2. The two groups successfully matched the variables, excluding age, fracture classification, and aCCI (*p* < 0.05). Therefore, these variables need to be adjusted in the multivariate analysis.

In the PSM crude and PSM adjusted models, we found the modifying effect of TTA on the ATO–mortality relationship remained significant (*p* for interaction = 0.0339 in crude model and 0.0346 in adjusted model), as shown in Supplementary Table S3. In the TTA ≤ 24 h subgroup, each one-day ATO delay remained associated with a 5% increase in mortality (HR = 1.05; 95% CI: 1.01–1.09, *p* = 0.0235 in crude model and HR = 1.05; 95% CI: 1.01–1.09, *p* = 0.0235 in adjusted model). In the TTA > 24 h subgroup, ATO delay remained not significantly associated with mortality (HR = 0.98; 95% CI: 0.93–1.03, *p* = 0.4413 in crude model and HR = 0.98; 95% CI: 0.94–1.03, *p* = 0.4471 in adjusted model).

### 3.6. The Stratified Result in Interaction Role of TTA on the Association Between ATO and Mortality

We performed a stratification analysis to test the stability of the interaction role of TTA, as shown in Table 5. The TTA–ATO–mortality interaction varies in high-risk populations. There was an interaction role of TTA on the association between ATO and mortality in the subgroups of female, femoral neck fracture, aCCI ≤ 4, present hypertension, present CHD, present arrhythmia, and absent COPD. The ATO delay was associated with a 6–13% increase in the mortality rate of patients in the TTA ≤ 24 h group. The result was close to 5% for the patients overall. In contrast, there was no difference between ischemic stroke and non-ischemic stroke.

## 4. Discussion

This retrospective cohort study is the first to demonstrate that TTA significantly modifies the association between ATO and long-term all-cause mortality among elderly patients with hip fractures. Our stratified analyses revealed divergent ATO-related mortality risks conditional on the 24 h TTA threshold: each one-day extension of ATO corresponded to a 5% higher mortality risk only in patients admitted within 24 h of injury, while no significant ATO–mortality association was observed for patients with TTA exceeding 24 h. Subgroup stratification further identified distinct high-risk populations where this TTA–ATO–mortality interaction was prominent, including females, patients with femoral neck fractures, and individuals with mild comorbidity burden (aCCI ≤ 4) complicated by hypertension, coronary heart disease, or arrhythmia, but without chronic obstructive pulmonary disease. These findings uncover a critical interplay between two perioperative time windows and provide evidence for stratified clinical decision-making for elderly hip fracture care. In the PSM analysis, the modifying effect of TTA on the ATO–mortality relationship remained significant, with a similar effect direction and magnitude as the primary analysis. However, it is important to note that despite matching, significant imbalances remained for age, fracture classification, and aCCI, indicating that the PSM algorithm did not achieve full covariate balance. Therefore, the PSM results should be interpreted as a secondary sensitivity analysis that generally supports the primary findings.

TTA refers to the period from injury to admission to the hospital, where subsequent surgical treatment is received. At its core is the delay occurring during the pre-hospital phase. It encompasses key steps: the patient or their family recognizing the injury, calling for emergency help, and transporting the patient to the hospital, where surgery will ultimately be performed. This phase can be affected by several factors, including the patient’s cognitive abilities (e.g., misjudging the severity of the injury), availability of family support (such as elderly individuals living alone with no assistance), and the efficiency of the emergency medical system (for instance, prolonged transport times in remote areas). While TTA serves as a time-window indicator, this temporal gap does not merely reflect disparities in medical resource distribution. Notably, TTA and ATO differ substantially: TTA reflects the efficiency of pre-hospital emergency care and social support, whereas ATO reflects a hospital’s internal resource allocation and clinical decision-making processes. An extended TTA directly exacerbates the patient’s pathophysiological response to acute trauma, increasing the risk of fracture-related complications and elevating surgical risks. Prolonged bed rest during this period may also lead to dehydration, pressure ulcers, lower limb venous thrombosis, and delirium due to inadequate pre-hospital pain management. In contrast, an extended ATO is associated with risks such as aggravated occult bleeding from perioperative anticoagulation, impaired functional recovery, further increased surgical risks, and higher medical costs. Thus, TTA and ATO represent distinct stages in the preoperative waiting period: the former highlights an “interface challenge” between the medical system and social support, while the latter reflects an “efficiency issue” in hospital resource allocation. We also observed that patients with longer TTA received faster surgery (ATO ≤ 2 d) than those with shorter TTA. This difference is explained by several interrelated factors. First, a substantial proportion (18.6%) of patients in the ATO ≤ 2 d group were transferred from other trauma centers, where initial preoperative examinations had already been completed. For these patients, the recorded TTA is prolonged because it includes the time spent at the referring hospital; however, upon arrival at our center, they could proceed directly to surgery without extensive preoperative evaluation, resulting in a short ATO. Second, among non-transferred patients in the ATO ≤ 2 d group, those with TTA > 24 h typically experienced pre-hospital delays but arrived in a clinically stable condition requiring no prolonged preoperative optimization, thus permitting expedited surgery. In contrast, the other ATO > 6 d groups are predominantly composed of patients with TTA ≤ 24 h who arrived early but required in-hospital medical optimization for acute comorbidities, which prolonged their ATO. These findings highlight that TTA and ATO represent distinct phases, pre-hospital and in-hospital, each with different determinants, and their combined effect on outcomes is nuanced rather than unidirectional. The observed pattern is also consistent with the prior literature documenting that transfer patients often have prolonged pre-hospital time but expedited in-hospital surgery [14].

In elderly hip fractures, the association between ATO and the mortality rate was notably complex. Early studies showed that surgery within 24 to 48 h might reduce mortality [29,30]. However, some subsequent studies failed to confirm the association between ATO delay and increased postoperative mortality [31,32,33,34]. Additionally, several investigations have explored how TTA correlates with outcomes in this population. In a retrospective study by He et al., they found 25.4% of patients were admitted on the day of injury, 54.7% within the first day, and 66.3% within the first two days; notably, 12.6% sought medical care as late as one week after injury [17]. A prospective cohort study of 78 patients identified a TTA delay of 3.45 d as a risk factor for 30-day mortality [35]. He et al. also found that admission delays exceeding one week were significantly associated with a 76% higher 1-year mortality [17]. In a larger study of 3893 hip fracture cases, indirect admission via patient transfer prolonged TTA, which in turn increased perioperative complications and mortality [36]. Additionally, Vidal et al. demonstrated that delayed TTA was linked to a 9% lower post-discharge survival rate and a 7% reduction in 1-year post-surgical survival [16]. Our previous study found that a delay in TTA was a risk factor for the 1-year mortality rate and the mortality rate during follow-up [24]. Notably, the relationship between TTA and the mortality rate was curvilinear. When TTA was less than 24 h, TTA was associated with the long-term mortality rate of elderly patients with hip fractures. When TTA exceeded 24 h, there was no association. Therefore, TTA is a very crucial indicator of the time window.

This study analyzed TTA, ATO, and the patient’s prognosis and discovered the modifying effect among them. Building on this TTA-driven modifying effect, it is suggested that the clinical strategy of shortening ATO should be implemented in combination with TTA stratification. For patients with TTA ≤ 24 h, minimizing ATO may be particularly important. In this subgroup, even a 1 h reduction in ATO may be significantly associated with meaningful improvements in patient outcomes. For patients with TTA > 24 h, completion of necessary examinations after admission and surgery as early as feasible following stabilization may be considered. Short-term interventions or multidisciplinary evaluation should be provided when necessary, and surgical treatment should proceed once the patient’s physical condition is optimized.

To optimize TTA, we recommend the following measures: (1) Launch community-based public education campaigns (e.g., posters, lectures) to raise awareness. (2) Train family members to recognize the classic triad of senile osteoporotic fractures, with specific emphasis on hip fractures. (3) Streamline emergency rescue protocols, ensuring priority dispatch of emergency resources to elderly patients suspected of fractures. (4) Equip ambulances with portable X-ray machines when possible to enable pre-hospital diagnosis and initiate surgical preparations early. (5) Integrate TTA into public health monitoring metrics and establish regional trauma networks. Implementing a TTA-oriented stratified surgical pathway comes with both feasibility and challenges. Its feasibility lies in TTA’s straightforward stratification logic, which is adaptable even for primary hospitals. Key challenges include resource constraints (e.g., insufficient surgical staff in primary settings, strained resource allocation in large hospitals with high emergency volumes) and pre-hospital barriers (e.g., inefficiencies in pre-hospital emergency systems, limited public awareness), which may diminish the impact of in-hospital interventions.

The potential mechanisms underlying TTA’s modifying effect on the relationship between ATO and mortality in elderly patients with hip fractures are as follows. In the TTA ≤ 24 h group, patients admitted early will likely be in acute stress. Delayed surgery, ATO delay, exacerbate systemic inflammatory responses and accelerate the development of fracture-related complications. This aligns with prior findings that hematologic inflammatory markers are independently associated with higher long-term mortality in older hip fracture patients [37,38], and that anti-inflammatory medications can reduce in-hospital complications [39]. The observed 5% increase in mortality for each 1-day ATO delay in this group underscores the critical importance of early intervention following trauma. In contrast, for the TTA > 24 h group, delayed admission may signal underlying issues such as inadequate social support, complex comorbidities, or poor physiological compensatory capacity. By this stage, the body has likely entered a state of chronic compensation, where the timing of surgery has a relatively diminished impact on overall physical status. Thus, 24 h post-injury may represent a critical threshold for the failure of physiological compensatory mechanisms: early-admitted patients require prompt anatomical restoration to halt pathological processes, whereas those with TTA > 24 h benefit more from comprehensive preoperative optimization. This mechanism is supported by clinical data: the median total waiting time (TTA + ATO) was 100 h in the TTA ≤ 24 h group, compared to 264 h in the TTA > 24 h group. Therefore, the difference between the two groups may mainly be due to the shorter overall waiting time for surgery in the TTA ≤ 24 h group, which results in a lower mortality rate.

Current guidelines emphasize a “24–48 h surgical window” [29]. However, our findings suggest that the TTA ≤ 24 h subgroup may require more stringent time management, and its supplementary value to guideline-based practice warrants further discussion. In fact, Panesar et al., summarizing the existing evidence, proposed that comorbidities and overall health status should stratify elderly patients. For those with poor general condition, priority should be given to optimizing their physiological status upon admission, including bringing chronic comorbidities as close to baseline as possible, before proceeding with urgent hip fracture repair [40]. Similarly, Goubar et al. developed the Stratify–Hip algorithm, which incorporates predictive models to classify patients into overall low-risk (low risk across all outcomes), medium-risk (low mortality risk but moderate-to-high risk of residence change), and high-risk (high risk of in-hospital death and high-to-moderate risk of 30-day mortality) categories [41]. This approach diverges from the traditional recommendation of expedited surgery via “green passage” for elderly patients with hip fractures. In this study, we suggest the following strategies based on TTA stratification. For patients with TTA ≤ 24 h, minimizing ATO may be particularly important. For patients with TTA > 24 h, completion of necessary examinations after admission and surgery as early as feasible following stabilization may be considered. This strategy could maximize the efficiency of medical resource utilization while improving patient outcomes. From a study perspective, a deeper exploration of chronobiological mechanisms is needed to advance toward precision trauma care. These insights offer a nuanced refinement of the traditional “the sooner the surgery, the better” paradigm. While the TTA-stratified approach holds theoretical promise, its clinical implementation presents practical challenges and ethical considerations. TTA is influenced by pre-hospital factors such as emergency medical service efficiency and social support-elements largely beyond hospital control, which cannot be fully compensated by an in-hospital “green passage”. Moreover, if TTA correlates with socioeconomic status, prioritizing patients with TTA ≤ 24 h may inadvertently exacerbate health inequities through a “cumulative advantage” effect. Surgeons should also avoid the potential pitfall of routinely assigning patients with TTA > 24 h to a “preoperative stabilization” strategy without individualized assessment, as this could introduce unintended disparities. Therefore, TTA stratification should be regarded as a reference for clinical decision-making rather than the sole determinant of resource allocation. Its application must be accompanied by efforts to strengthen pre-hospital emergency systems and optimize resource distribution, ensuring that all patients receive equitable, needs-based care.

The heterogeneity of the TTA-modifying effect across specific subgroups revealed by our analysis provides deeper insights into its underlying mechanisms and potential for precise clinical application. The significant interaction observed in female patients, but not in males, may be attributed to the confluence of greater physiological frailty and distinct sociodemographic factors in elderly women with hip fractures. Physiologically, older women generally present with lower skeletal muscle mass and bone density, potentially leading to a more limited reserve for physiological compensation after acute trauma. This could render them more susceptible to the detrimental effects of ATO delay, potentially leading to an accelerated cascade of inflammatory responses and complications [42]. From a sociodemographic perspective, TTA may serve as a proxy for the timeliness of social support networks. Older women, particularly those living alone, may face greater challenges in this regard. Consequently, a “TTA ≤ 24 h” might identify a subgroup of women who received prompt assistance, making ATO delay the critical controllable determinant of their outcome. For women with prolonged TTA likely due to insufficient social support, their prognosis may already be predominantly shaped by pre-admission adversities, thereby attenuating the observable impact of ATO. Secondly, the effect modification was significant in patients with a lower comorbidity burden (aCCI ≤ 4) but not in those with a higher burden (aCCI > 4). This discrepancy is likely explained by “competing risks.” For patients with multiple severe comorbidities, mortality is primarily driven by these underlying conditions (e.g., advanced heart or renal failure), leaving limited room for improvement from a single factor like ATO, whose effect can be masked by the overpowering baseline risk [42]. Furthermore, clinical practice inherently delays surgery in these complex patients for optimization, making ATO more a marker of case complexity than an independent intervention target. In contrast, for relatively healthier patients (aCCI ≤ 4), survival outcomes are more directly influenced by the fracture and its complication trajectory. Thus, the entire timeline from injury to surgery, particularly ATO delay, becomes a key modifiable factor. TTA acts as a stratifier: expediting surgery is crucial for early presenters, while comprehensive preoperative optimization becomes paramount for late presenters. However, these findings suggest that the modifying effect of TTA is most pronounced in patient populations with a more “plastic” baseline risk profile and less dominated by competing terminal conditions, such as relatively healthier individuals or specific gender groups. These subgroup disparities provide pathophysiological and socioepidemiological evidence supporting stratified, timing-targeted clinical management for elderly hip fracture patients. Importantly, these subgroup findings were derived from exploratory analyses without adjustment for multiple testing. Given the number of subgroup comparisons performed, some statistically significant findings may have occurred by chance. Thus, these results should be considered exploratory and require independent validation before any clinical conclusions can be drawn.

Several limitations of this study should be acknowledged. First, residual confounding and confounding by indication may persist despite multivariable adjustment and PSM. Patients with ATO delay exhibited worse baseline comorbidity profiles, and ATO delay was largely driven by pre-existing severe illness that independently elevated mortality risk. Unmeasured factors, including surgical schedule constraints, hospital resource availability, patient frailty, cognitive dysfunction, and long-term home care quality, could not be incorporated into our regression models, which may weaken causal inference regarding the independent effect of ATO. Second, all-cause mortality was selected as the primary endpoint, thereby preventing us from distinguishing disease-specific mortality pathways mediated by perioperative complications, thromboembolism, or cardiovascular decompensation; future prospective studies should collect cause-of-death data to validate underlying mechanisms. Third, TTA data were extracted from medical records relying on patient and family self-report, introducing potential non-differential measurement error that may underestimate the true magnitude of effect modification. Fourth, this single-center retrospective cohort may limit generalizability to other trauma systems with disparate pre-hospital emergency workflows. Finally, all subgroup interaction analyses were exploratory without correction for multiple testing; significant subgroup findings should be interpreted as hypothesis-generating and require external validation in independent cohorts.

## 5. Conclusions

In conclusion, TTA is an important effect modifier of the association between ATO and mortality in elderly patients with hip fractures.

## Figures and Tables

**Table 1:** Demographic and clinical characteristics.

ATO	ATO ≤ 2 d	2 d < ATO ≤ 4 d	4 d < ATO ≤ 6 d	ATO > 6 d	*p*-Value	*p*-Value *
N	556	976	512	316		
ATO (d)	1.89 ± 0.31	3.50 ± 0.50	5.41 ± 0.49	9.12 ± 3.23	<0.001	<0.001
Age (y)	79.47 ± 6.68	79.54 ± 6.65	79.13 ± 6.74	79.57 ± 6.80	0.685	0.755
Sex					0.651	-
Male	166 (29.86%)	304 (31.15%)	171 (33.40%)	101 (31.96%)		
Female	390 (70.14%)	672 (68.85%)	341 (66.60%)	215 (68.04%)		
Injury mechanism					0.312	-
Falling	540 (97.12%)	949 (97.23%)	497 (97.07%)	299 (94.62%)		
Accident	12 (2.16%)	23 (2.36%)	13 (2.54%)	13 (4.11%)		
Other	4 (0.72%)	4 (0.41%)	2 (0.39%)	4 (1.27%)		
Fracture classification					0.123	-
Intertrochanteric fracture	396 (71.22%)	713 (73.05%)	392 (76.56%)	243 (76.90%)		
Femoral neck fracture	160 (28.78%)	263 (26.95%)	120 (23.44%)	73 (23.10%)		
aCCI	4.06 ± 1.03	4.14 ± 1.03	4.23 ± 1.11	4.35 ± 1.08	<0.001	<0.001
Hypertension					0.116	-
No	302 (54.32%)	504 (51.64%)	254 (49.61%)	146 (46.20%)		
Yes	254 (45.68%)	472 (48.36%)	258 (50.39%)	170 (53.80%)		
Diabetes					0.012	-
No	467 (83.99%)	796 (81.56%)	397 (77.54%)	242 (76.58%)		
Yes	89 (16.01%)	180 (18.44%)	115 (22.46%)	74 (23.42%)		
CHD					<0.001	-
No	292 (52.52%)	506 (51.84%)	224 (43.75%)	123 (38.92%)		
Yes	264 (47.48%)	470 (48.16%)	288 (56.25%)	193 (61.08%)		
Arrhythmia					<0.001	-
No	401 (72.12%)	698 (71.52%)	329 (64.26%)	175 (55.38%)		
Yes	155 (27.88%)	278 (28.48%)	183 (35.74%)	141 (44.62%)		
Hemorrhagic stroke					0.731	-
No	545 (98.02%)	957 (98.05%)	503 (98.24%)	307 (97.15%)		
Yes	11 (1.98%)	19 (1.95%)	9 (1.76%)	9 (2.85%)		
Ischemic stroke					0.088	-
No	412 (74.10%)	710 (72.75%)	363 (70.90%)	210 (66.46%)		
Yes	144 (25.90%)	266 (27.25%)	149 (29.10%)	106 (33.54%)		
Cancer					0.318	-
No	545 (98.02%)	950 (97.34%)	497 (97.07%)	303 (95.89%)		
Yes	11 (1.98%)	26 (2.66%)	15 (2.93%)	13 (4.11%)		
Associated injuries					0.788	-
No	520 (93.53%)	903 (92.52%)	477 (93.16%)	297 (93.99%)		
Yes	36 (6.47%)	73 (7.48%)	35 (6.84%)	19 (6.01%)		
Dementia					0.492	-
No	537 (96.58%)	944 (96.72%)	489 (95.51%)	301 (95.25%)		
Yes	19 (3.42%)	32 (3.28%)	23 (4.49%)	15 (4.75%)		
COPD					<0.001	-
No	537 (96.58%)	928 (95.08%)	467 (91.21%)	291 (92.09%)		
Yes	19 (3.42%)	48 (4.92%)	45 (8.79%)	25 (7.91%)		
Hepatitis					0.362	-
No	540 (97.12%)	952 (97.54%)	491 (95.90%)	307 (97.15%)		
Yes	16 (2.88%)	24 (2.46%)	21 (4.10%)	9 (2.85%)		
Gastritis					0.089	-
No	550 (98.92%)	956 (97.95%)	506 (98.83%)	306 (96.84%)		
Yes	6 (1.08%)	20 (2.05%)	6 (1.17%)	10 (3.16%)		
Albumin (g/L)	38.14 ± 3.85	38.06 ± 3.93	37.86 ± 3.96	37.13 ± 4.07	0.003	0.009
TTA (h) ^†^	24 (4–168)	8 (4–48)	10.5 (4–48)	10 (4–48)	<0.001	<0.001
Treatment strategy					0.085	-
CRIF/ORIF	388 (69.78%)	711 (72.85%)	388 (75.78%)	236 (74.68%)		
HA	164 (29.50%)	245 (25.10%)	117 (22.85%)	76 (24.05%)		
THA	4 (0.72%)	20 (2.05%)	7 (1.37%)	4 (1.27%)		
Operation time (mins) ^†^	80 (60–100)	90 (70–110)	90 (70–120)	90 (70–110)	<0.001	<0.001
Blood loss (mL) ^†^	200 (150–300)	200 (150–300)	200 (150–300)	200 (150–300)	0.084	0.516
Infusion (mL) ^†^	1600 (1100–1600)	1600 (1200–1600)	1600 (1325–1600)	1600 (1125–1600)	0.029	0.099
Transfusion (U) ^†^	0 (0–2)	0 (0–2)	2 (0–2)	2 (0–2)	0.001	0.001
Follow-up (m) ^†^	37.42 (29.64–47.19)	39.47 (29.24–51.33)	41.30 (27.98–56.67)	38.35 (28.47–50.99)	0.025	0.033
Mortality					0.013	-
Survival	383 (68.88%)	703 (72.03%)	342 (66.80%)	199 (62.97%)		
Dead	173 (31.12%)	273 (27.97%)	170 (33.20%)	117 (37.03%)		
Mortality in TTA group						
TTA ≤ 24 h	72 (32.29%)	194 (27.29%)	133 (33.33%)	105 (38.04%)		
TTA > 24 h	101 (30.33%)	79 (29.81%)	37 (32.74%)	12 (30.00%)		

Mean + SD/N (%). ^†^ Median with IQR. *p*-value *: for continuous variables, we used the Kruskal–Wallis rank–sum test and Fisher’s exact probability test for count variables with a theoretical number of <10.

**Table 2:** Results of the univariate Cox regression analysis of the relationship between each variable and the mortality during long-term follow-up.

Risk Factors	Statistics	HR (95% CI)	*p*-Value
Age (y)	79.44 ± 6.70	1.08 (1.06, 1.09)	<0.0001
Sex			
Male	742 (31.44%)	1	
Female	1618 (68.56%)	0.73 (0.63, 0.85)	<0.0001
Injury mechanism			
Falling	2285 (96.82%)	1	
Accident	61 (2.58%)	0.30 (0.14, 0.64)	0.0018
Other	14 (0.59%)	1.49 (0.67, 3.32)	0.3326
Fracture classification			
Intertrochanteric fracture	1744 (73.90%)	1	
Femoral neck fracture	616 (26.10%)	0.98 (0.82, 1.18)	0.8458
aCCI	4.17 ± 1.06	1.49 (1.39, 1.59)	<0.0001
Hypertension	1154 (48.90%)	1.11 (0.96, 1.28)	0.1691
Diabetes	458 (19.41%)	1.02 (0.85, 1.23)	0.8339
CHD	1215 (51.48%)	1.20 (1.04, 1.39)	0.0138
Arrhythmia	757 (32.08%)	1.21 (1.04, 1.40)	0.0151
Hemorrhagic stroke	48 (2.03%)	0.95 (0.56, 1.61)	0.8421
Dementia	89 (3.77%)	2.54 (1.91, 3.37)	<0.0001
Ischemic stroke	665 (28.18%)	1.36 (1.16, 1.59)	0.0001
Cancer	65 (2.75%)	1.66 (1.15, 2.39)	0.0067
Associated injuries	163 (6.91%)	0.86 (0.64, 1.17)	0.345
COPD	137 (5.81%)	1.47 (1.12, 1.93)	0.0057
Hepatitis	70 (2.97%)	1.48 (1.03, 2.14)	0.0347
Gastritis	42 (1.78%)	0.83 (0.47, 1.46)	0.5099
Albumin (g/L)	37.91 ± 3.95	0.91 (0.89, 0.93)	<0.0001
TTA (h) ^†^	10 (4–48)	1.00 (1.00, 1.00)	0.0094
ATO (d)	4.29 ± 2.57	1.02 (0.99, 1.05)	0.1431
Treatment strategy			
CRIF/ORIF	1723 (73.01%)	1	
HA	602 (25.51%)	1.08 (0.91, 1.29)	0.3906
THA	35 (1.48%)	0.20 (0.05, 0.82)	0.0248
Operation time (mins)	93.09 ± 35.95	1.00 (1.00, 1.00)	0.1524
Blood loss (mL)	241.44 ± 153.60	1.00 (1.00, 1.00)	0.5341
Infusion (mL)	1556.11 ± 383.07	1.00 (1.00, 1.00)	0.0002
Transfusion (U)	1.12 ± 1.24	1.05 (0.99, 1.11)	0.0876

^†^ Median with IQR.

**Table 3:** Multivariate results by Cox regression.

Exposed Variables	Non-Adjusted Model	Minimally Adjusted Model	Fully Adjusted Model
ATO	1.02 (0.99, 1.05) 0.1431	1.02 (0.99, 1.04) 0.1541	1.01 (0.99, 1.04) 0.3134
ATO subgroups			
ATO ≤ 2 d	1	1	1
2 d < ATO ≤ 4 d	0.85 (0.70, 1.03) 0.0935	0.83 (0.69, 1.01) 0.0608	0.88 (0.72, 1.09) 0.2408
4 d < ATO ≤ 6 d	0.98 (0.79, 1.21) 0.8223	0.99 (0.80, 1.23) 0.9333	0.93 (0.73, 1.18) 0.5401
ATO > 6 d	1.15 (0.91, 1.46) 0.2347	1.14 (0.90, 1.45) 0.2645	1.10 (0.85, 1.43) 0.4512
*p* for trend	0.1543	0.1325	0.4346

Data in the table: HR (95% CI) *p*-value; outcome variable: mortality; exposed variables: ATO; minimally adjust for age, sex. The fully adjusted model was adjusted for age, sex, aCCI, albumin, transfusion, and infusion.

**Table 4:** Analysis of the interaction effects on the association between ATO and mortality.

Interaction Factors	Unadjusted Model			Adjusted Model		
	N	HR (95% CI) *p*-Value	Interaction *p*-Value	N	HR (95% CI) *p*-Value	Interaction *p*-Value
Sex						
Male	742	1.01 (0.97, 1.04) 0.7380	0.4121	723	0.99 (0.95, 1.04) 0.7367	0.1299
Female	1618	1.03 (0.99, 1.07) 0.1489		1565	1.04 (1.00, 1.08) 0.0694	
aCCI						
≤4	1568	1.02 (0.98, 1.05) 0.3014	0.7025	1528	1.02 (0.99, 1.06) 0.1701	0.4856
>4	792	1.01 (0.97, 1.05) 0.7090		760	1.00 (0.96, 1.05) 0.9254	
TTA (h)						
≤24 h	1649	1.06 (1.02, 1.10) 0.0012	0.0036	1607	1.05 (1.01, 1.09) 0.0095	0.0109
>24 h	711	0.98 (0.94, 1.02) 0.3632		681	0.98 (0.93, 1.02) 0.3050	
Hypertension						
Yes	1154	1.03 (0.99, 1.06) 0.1031	0.4123	1121	1.01 (0.98, 1.05) 0.4047	0.9302
No	1206	1.00 (0.96, 1.05) 0.8256		1167	1.01 (0.97, 1.06) 0.6073	
CHD						
Yes	1215	1.01 (0.98, 1.05) 0.4969	0.6969	1175	1.01 (0.97, 1.05) 0.7296	0.6638
No	1145	1.02 (0.98, 1.06) 0.2610		1113	1.02 (0.98, 1.06) 0.3081	
Arrhythmia						
Yes	757	1.00 (0.96, 1.05) 0.8410	0.4825	737	1.01 (0.96, 1.06) 0.7004	0.7704
No	1603	1.02 (0.99, 1.06) 0.1663		1551	1.02 (0.99, 1.05) 0.2793	
Ischemic stroke						
Yes	665	1.03 (0.99, 1.08) 0.1284	0.3737	644	1.04 (0.98, 1.10) 0.1733	0.3336
No	1695	1.01 (0.98, 1.04) 0.5982		1644	1.01 (0.97, 1.04) 0.7063	
COPD						
Yes	137	0.98 (0.89, 1.08) 0.6631	0.4142	131	0.99 (0.88, 1.13) 0.9313	0.7432
No	2223	1.02 (0.99, 1.05) 0.1416		2157	1.02 (0.99, 1.05) 0.2678	

Data in the table: HR (95% CI) *p*-value; outcome variable: mortality; exposed variables: ATO. The model was adjusted for age, sex, aCCI, albumin, transfusion, and infusion.

**Table 5:** Stratified analysis of the interaction effects on the association between ATO and mortality.

Stratified Analysis	TTA ≤ 24 h HR (95% CI) *p*-Value	TTA > 24 h HR (95% CI) *p*-Value	Interaction *p*-Value
Sex			
Male	1.02 (0.95, 1.09) 0.5572	0.97 (0.90, 1.03) 0.2971	0.2374
Female	1.07 (1.02, 1.12) 0.0056	0.98 (0.91, 1.06) 0.6727	0.0426
Fracture classification			
Intertrochanteric fracture	1.04 (0.99, 1.08) 0.1175	0.97 (0.92, 1.03) 0.3569	0.0796
Femoral neck fracture	1.13 (1.03, 1.23) 0.0081	0.99 (0.91, 1.07) 0.7737	0.0300
aCCI			
≤4	1.09 (1.03, 1.14) 0.0021	0.98 (0.93, 1.04) 0.5423	0.0071
>4	1.03 (0.97, 1.09) 0.3539	0.97 (0.89, 1.04) 0.3830	0.2072
Hypertension			
Yes	1.06 (1.01, 1.12) 0.0272	0.98 (0.93, 1.04) 0.5291	0.0441
No	1.05 (0.99, 1.12) 0.0876	0.97 (0.89, 1.06) 0.4756	0.1024
CHD			
Yes	1.05 (1.00, 1.10) 0.0586	0.94 (0.87, 1.01) 0.0966	0.0113
No	1.06 (0.99, 1.13) 0.0971	1.00 (0.95, 1.05) 0.9936	0.1932
Arrhythmia			
Yes	1.06 (1.00, 1.13) 0.0346	0.91 (0.82, 1.00) 0.0546	0.0041
No	1.05 (0.99, 1.11) 0.0893	1.01 (0.96, 1.05) 0.7882	0.2523
Ischemic stroke			
Yes	1.08 (1.01, 1.15) 0.0203	0.97 (0.87, 1.08) 0.5500	0.0836
No	1.05 (1.00, 1.10) 0.0746	0.98 (0.93, 1.03) 0.4843	0.0722
COPD			
Yes	1.00 (0.87, 1.15) 0.9940	0.88 (0.64, 1.22) 0.4461	0.4731
No	1.06 (1.02, 1.10) 0.0070	0.98 (0.93, 1.03) 0.3873	0.0121

Data in the table: HR (95% CI) *p*-value; outcome variable: mortality; exposed variables: ATO. The model was adjusted for age, sex, aCCI, albumin, transfusion, and infusion.

## Data Availability

Xi’an Honghui Hospital provided the data. According to relevant regulations, the data cannot be shared but may be requested from the corresponding author.

## Supplementary Materials

The following supporting information can be downloaded at: https://www.mdpi.com/article/10.3390/jcm15145685/s1, Table S1: Sensitivity Analysis: Comparison of the Modifying Effect of TTA on the ATO-Mortality Association Using Different Time Origins; Table S2: The balance test of PSM; Table S3: The modifying effect of TTA on the ATO-mortality under PSM; Figure S1: A Cox model with restricted cubic spline plot illustrating the relationship between TTA and the risk of long-term mortality; Figure S2: The distribution of propensity scores after PSM.

## References

[1] Tang V.L., Sudore R., Cenzer I.S., Boscardin W.J., Smith A., Ritchie C., Wallhagen M., Finlayson E., Petrillo L., Covinsky K. (2017). Rates of Recovery to Pre-Fracture Function in Older Persons with Hip Fracture: An Observational Study. J. Gen. Intern. Med..

[2] O’Connor M.I., Switzer J.A. (2022). AAOS Clinical Practice Guideline Summary: Management of Hip Fractures in Older Adults. J. Am. Acad. Orthop. Surg..

[3] (2023). Hip Fracture: Management.

[4] Walter N., Szymski D., Kurtz S.M., Lowenberg D.W., Alt V., Lau E.C., Rupp M. (2023). Epidemiology and treatment of proximal femoral fractures in the elderly U.S. population. Sci. Rep..

[5] Griffiths R., Babu S., Dixon P., Freeman N., Hurford D., Kelleher E., Moppett I., Ray D., Sahota O., Shields M. (2021). Guideline for the management of hip fractures 2020: Guideline by the Association of Anaesthetists. Anaesthesia.

[6] Bhatti U.F., Shah A.A., Williams A.M., Biesterveld B.E., Okafor C., Ilahi O.N., Alam H.B. (2021). Delay in Hip Fracture Repair in the Elderly: A Missed Opportunity Towards Achieving Better Outcomes. J. Surg. Res..

[7] Murphy J.R., Loh J., Smith N.C., Stone N.C. (2022). Association of length of hospital stay with delay to surgical fixation of hip fracture. Can. J. Surg..

[8] Castillón P., Nuñez J.H., Mori-Gamarra F., Ojeda-Thies C., Sáez-López P., Salvador J., Anglés F., González-Montalvo J.I., Rnfc P.I.T. (2021). Hip fractures in Spain: Are we on the right track? Statistically significant differences in hip fracture management between Autonomous Communities in Spain. Arch. Osteoporos..

[9] Su S., Zhang Y., Wang R., Zhou R., Chen Z., Zhou F. (2023). Early surgery within 48 h was associated with reduced perioperative blood loss and red blood cell transfusion requirements in older patients with hip fracture: A retrospective study. Eur. Geriatr. Med..

[10] Pincus D., Ravi B., Wasserstein D., Huang A., Paterson J.M., Nathens A.B., Kreder H.J., Jenkinson R.J., Wodchis W.P. (2017). Association Between Wait Time and 30-Day Mortality in Adults Undergoing Hip Fracture Surgery. JAMA.

[11] Mitchell S.M., Chung A.S., Walker J.B., Hustedt J.W., Russell G.V., Jones C.B. (2018). Delay in Hip Fracture Surgery Prolongs Postoperative Hospital Length of Stay but Does Not Adversely Affect Outcomes at 30 Days. J. Orthop. Trauma.

[12] Ogawa T., Aoki T., Shirasawa S. (2019). Effect of hip fracture surgery within 24 hours on short-term mobility. J. Orthop. Sci..

[13] Warren M., Bretherton C., Parker M. (2024). Delay to surgery beyond 12 hours is associated with increased hip fracture mortality. Eur. J. Orthop. Surg. Traumatol..

[14] Armstrong E., Rogers K., Li C.S., Jagnoor J., Moroz P., Oguzie G.C., Hailu S., Miclau T., de la Huerta F., Martinez-Ruiz J.d.J. (2024). Time from injury to hip-fracture surgery in low-income and middle-income regions: A secondary analysis of data from the International Orthopaedic Multicentre Study in Fracture Care (INORMUS). Lancet Healthy Longev..

[15] The HIP ATTACK Investigators (2020). Accelerated surgery versus standard care in hip fracture (HIP ATTACK): An international, randomised, controlled trial. Lancet.

[16] Vidal E.I.O., Moreira-Filho D.C., Pinheiro R.S., Souza R.C., Almeida L.M., Camargo K.R., Boas P.J.F.V., Fukushima F.B., Coeli C.M. (2012). Delay from fracture to hospital admission: A new risk factor for hip fracture mortality?. Osteoporos. Int..

[17] He W., You Y.-Y., Sun K., Xie C., Ming Y., Yu L.-N., Zhang F.-J., Yan M. (2020). Admission delay is associated with worse surgical outcomes for elderly hip fracture patients: A retrospective observational study. World J. Emerg. Med..

[18] Asano Y., Takasugi T., Ueno K., Kondo N., Yoshino A., Ojima T. (2024). Association between social support and ambulance use among older people in Japan: An empirical cross-sectional study. BMC Emerg. Med..

[19] Moonesar R., Sammy I., Nunes P., Paul J. (2016). Social support in older people: Lessons from a developing country. Qual. Life Res..

[20] Luo W., Yao J., Mitchell R., Zhang X., Li W. (2022). Locating emergency medical services to reduce urban-rural inequalities. Socio-Econ. Plan. Sci..

[21] Zhang Y., Kolam I., Tumin D. (2025). Preinjury functional status is associated with functional status after hip fracture in older adults without preinjury perceived social support. Int. J. Rehabil. Res..

[22] Rashid R., Sohrabi C., Kerwan A., Franchi T., Mathew G., Nicola M., Agha R.A. (2024). The STROCSS 2024 guideline: Strengthening the reporting of cohort, cross-sectional, and case-control studies in surgery. J. Int. J. Surg..

[23] Agha R.A., Mathew G., Rashid R., Kerwan A., Al-Jabir A., Sohrabi C., Franchi T., Nicola M., Agha M. (2025). Revised Strengthening the reporting of cohort, cross-sectional and case-control studies in surgery (STROCSS) Guideline: An update for the age of Artificial Intelligence. Prem. J. Sci..

[24] Zhang B.-F., Xu S.-L., Yang Z., Xu P. (2024). Early admission is better-the time to admission (TTA) is associated with one-year mortality in hip fracture. Int. J. Surg..

[25] Zhang D.-L., Cong Y.-X., Zhuang Y., Xu X., Zhang B.-F. (2023). Age-adjusted Charlson comorbidity index predicts postoperative mortality in elderly patients with hip fracture: A prospective cohort. Front. Med..

[26] Huang H., Liu Y., Zhang B.F. (2025). Elevated albumin: A protective factor against mortality in geriatric hip fracture patients. J. Orthop. Surg. Res..

[27] Hori K., Siu A.M., Nguyen E.T., Andrews S.N., Choi S.Y., Ahn H.J., Nakasone C.K., Lim S.Y. (2020). Osteoporotic hip fracture mortality and associated factors in Hawaii. Arch. Osteoporos..

[28] Zhang B.F., Wang M.X. (2026). Threshold Effect of Time to Admission on Long-Term Mortality in Geriatric Hip Fractures: A 24-H Critical Window Identified. J. Clin. Med..

[29] Klestil T., Röder C., Stotter C., Winkler B., Nehrer S., Lutz M., Klerings I., Wagner G., Gartlehner G., Nussbaumer-Streit B. (2018). Impact of timing of surgery in elderly hip fracture patients: A systematic review and meta-analysis. Sci. Rep..

[30] Welford P., Jones C.S., Davies G., Kunutsor S.K., Costa M.L., Sayers A., Whitehouse M.R. (2021). The association between surgical fixation of hip fractures within 24 hours and mortality: A systematic review and meta-analysis. Bone Jt. J..

[31] de Palma L., Torcianti M., Meco L., Catalani A., Marinelli M. (2014). Operative delay and mortality in elderly patients with hip fracture: An observational study. Eur. J. Orthop. Surg. Traumatol..

[32] Ng M.K., Pasternack J.B., Mastrokostas P.G., Voyvodic L., Kang K.K. (2024). The real time to surgery: Limited delay after medical optimization does not impact hip fracture surgery outcomes. Injury.

[33] Wong S.H.J., Fang X.C., Yee K.H.D., Wong T.M., Pun C.T.T., Lau T.W., Leung K.L.F. (2018). Hip fracture time-to-surgery and mortality revisited: Mitigating comorbidity confounding by effect of holidays on surgical timing. Int. Orthop..

[34] Kawai M., Tanji A., Nishijima T., Tateyama K., Yoda Y., Iizuka A., Kamata Y., Urabe T. (2018). Association between time to surgery and 90-day mortality after hip fracture: A retrospective cohort study of 1734 cases. J. Orthop. Sci..

[35] Ghosh A.K., Patel S., Chouhan D., Samra T., Kanojia R.K., Bhalla A. (2023). Pre-Hospital Delays Represent Unnoticed Intervals That Affect Mortality Rates in Geriatric Hip Fractures: A Prospective Cohort Study. Cureus.

[36] Hughes A.J., Brent L., Biesma R., Kenny P.J., Hurson C.J. (2019). The effect of indirect admission via hospital transfer on hip fracture patients in Ireland. Ir. J. Med. Sci..

[37] Pan W.-G., Chou Y.-C., Wu J.-L., Yeh T.-T. (2024). Impact of hematologic inflammatory markers on the prognosis of geriatric hip fracture: A systematic review and meta-analysis. Eur. J. Med. Res..

[38] Chen Y., Tu C., Liu G., Peng W., Zhang J., Ge Y., Tan Z., Bei M., Gao F., Tian M. (2024). Association between admission inflammatory indicators and 3-year mortality risk in geriatric patients after hip fracture surgery: A retrospective cohort study. Front. Surg..

[39] Hatano M., Sasabuchi Y., Ishikura H., Watanabe H., Tanaka T., Tanaka S., Yasunaga H. (2024). Outcomes after hip fracture surgery in patients receiving non-steroidal anti-inflammatory drugs alone, acetaminophen alone, or both. Bone Jt. J..

[40] Panesar S.S., Simunovic N., Bhandari M. (2012). When should we operate on elderly patients with a hip fracture? It’s about time!. Surgeon.

[41] Goubar A., Martin F.C., Sackley C., Foster N.E., Ayis S., Gregson C.L., Cameron I.D., Walsh N.E., Sheehan K.J. (2023). Development and Validation of Multivariable Prediction Models for In-Hospital Death, 30-Day Death, and Change in Residence After Hip Fracture Surgery and the “Stratify-Hip” Algorithm. J. Gerontol. Ser. A Biol. Sci. Med. Sci..

[42] Grigoryan K.V., Javedan H., Rudolph J.L. (2014). Orthogeriatric care models and outcomes in hip fracture patients: A systematic review and meta-analysis. J. Orthop. Trauma..

