# Supplementary material for: The 24-Hour Window: How Time to Admission Reshapes the Impact of Surgical Delay on Mortality in Elderly Hip Fracture
Source: J Clin Med. 2026 Jul 20;15(14):5685. doi: 10.3390/jcm15145685 (PMC13413047; doi:10.3390/jcm15145685)
Supplement: Supplementary file 1 [file jcm-15-05685-s001.zip › jcm-4407243-supplementary.pdf]

Supplementary Table S1. Sensitivity Analysis: Comparison of the Modifying Effect of TTA on the ATO-Mortality Association Using Different Time Origins

Subgroup	Analysis with Surgery Time as Time Zero (Original Analysis)		Analysis with Admission Time as Time Zero (Sensitivity Analysis)	
	HR (95% CI)	*P*-value	HR (95% CI)	*P*-value
**Crude model**				
**TTA≤24 hours**	1.0605 (1.0234, 1.0990)	0.0012	1.0596 (1.0225, 1.0980)	0.0015
**TTA>24 hours**	0.9816 (0.9432, 1.0217)	0.3632	0.9809 (0.9424, 1.0209)	0.3438
***P* for interaction**	0.0036		0.0036	
**Adjusted model**				
**TTA≤24 hours**	1.0524 (1.0126, 1.0939)	0.0095	1.0513 (1.0115, 1.0927)	0.0111
**TTA>24 hours**	0.9754 (0.9299, 1.0230)	0.3050	0.9745 (0.9291, 1.0221)	0.2889
***P* for interaction**	0.0109		0.0112	

The model was adjusted for age, sex, aCCI, albumin, transfusion, and infusion.

Supplementary Table S2. The balance test of PSM

Variables	Survival (N=600)	Dead (N=600)	Standardized diff.	*P* value
**ATO (d)** †	4 (3-5)	4 (3-5)	0.09 (-0.03, 0.20)	0.138
**Age (y)**	82.94 ± 4.96	81.93 ± 6.33	0.18 (0.06, 0.29)	0.002*
**Sex**			0.04 (-0.07, 0.15)	0.473
Male	217 (36.2)	229 (38.2)		
Female	383 (63.8)	371 (61.8)		
**Injury mechanism**			0.12 (0.00, 0.23)	0.135
Falling	586 (97.7)	588 (98)		
Accident	12 (2)	6 (1)		
Other	2 (0.3)	6 (1)		
**Fracture classification**			0.11 (0.00, 0.23)	0.047*
Intertrochanteric fracture	432 (72)	462 (77)		
Femoral neck fracture	168 (28)	138 (23)		
**aCCI**	5 (4-5)	4 (4-5)	0.13 (0.02, 0.24)	0.025*
**Hypertension**			0.07 (-0.04, 0.19)	0.204
No	286 (47.7)	308 (51.3)		
Yes	314 (52.3)	292 (48.7)		
**Diabetes**			0.07 (-0.04, 0.18)	0.226
No	467 (77.8)	484 (80.7)		
Yes	133 (22.2)	116 (19.3)		
**CHD**			0.01 (-0.11, 0.12)	0.908
No	275 (45.8)	273 (45.5)		
Yes	325 (54.2)	327 (54.5)		
**Arrhythmia**			0.04 (-0.08, 0.15)	0.54
No	405 (67.5)	395 (65.8)		
Yes	195 (32.5)	205 (34.2)		
**Hemorrhagic stroke**			0.01 (-0.10, 0.13)	0.817
No	590 (98.3)	591 (98.5)		
Yes	10 (1.7)	9 (1.5)		
**Ischemic stroke**			0.01 (-0.11, 0.12)	0.902
No	406 (67.7)	404 (67.3)		
Yes	194 (32.3)	196 (32.7)		
**Cancer**			0.04 (-0.07, 0.16)	0.466
No	578 (96.3)	573 (95.5)		
Yes	22 (3.7)	27 (4.5)		
**Associated injuries**			0.05 (-0.06, 0.16)	0.385
No	562 (93.7)	569 (94.8)		
Yes	38 (6.3)	31 (5.2)		
**Dementia**			0.09 (-0.02, 0.21)	0.112
No	571 (95.2)	558 (93)		
Yes	29 (4.8)	42 (7)		
**COPD**			0.02 (-0.09, 0.13)	0.738
No	558 (93)	555 (92.5)		
Yes	42 (7)	45 (7.5)		
**Hepatitis**			0.00 (-0.11, 0.11)	1
No	577 (96.2)	577 (96.2)		
Yes	23 (3.8)	23 (3.8)		
**Gastritis**			0.01 (-0.10, 0.13)	0.833

No	588 (98)	589 (98.2)		
Yes	12 (2)	11 (1.8)		
Albumin (g/L)	36.69 ± 3.57	36.73 ± 3.81	0.01 (-0.10, 0.12)	0.863
**TTA (hours)**†	12 (4-72)	17 (4-72)	0.04 (-0.08, 0.15)	0.512
**Treatment strategy**			0.12 (0.01, 0.23)	0.112
CRIF/ORIF	423 (70.5)	455 (75.8)		
HA	175 (29.2)	143 (23.8)		
THA	2 (0.3)	2 (0.3)		
**Operation time (mins)** †	90.00 (70-110)	90.00 (70-105)	0.02 (-0.10, 0.13)	0.783
**Blood loss (mL)** †	200 (150-300)	200 (150-300)	0.01 (-0.11, 0.12)	0.918
**Infusion (mL)** †	1600 (1100-1600)	1600 (1100-1600)	0.02 (-0.09, 0.13)	0.726
**Transfusion (U)** †	2 (0-2)	2 (0-2)	0.01 (-0.11, 0.12)	0.926
**Follow-up (m)** †	42.99 (35.21-53.66)	21.18 (9.39-32.54)	1.58 (1.45, 1.71)	<0.001

†Median with IQR.

*** Variables were not successfully matched.**

Supplementary Table S3. The modifying effect of TTA on the ATO-mortality under PSM

Model	TTA≤ 24 h	TTA> 24 h	*P* for interaction
No. of patients	810	390	
Crude model	1.05 (1.01, 1.09) 0.0235	0.98 (0.93, 1.03) 0.4413	0.0339
Adjusted model	1.05 (1.01, 1.09) 0.0235	0.98 (0.94, 1.03) 0.4471	0.0346

Data in table: HR (95% CI) *P*-value

Outcome variable: Mortality

Exposed variables: ATO

Modifying factor: TTA

Adjusted variables in the adjusted model: age, fracture classification and aCCI.

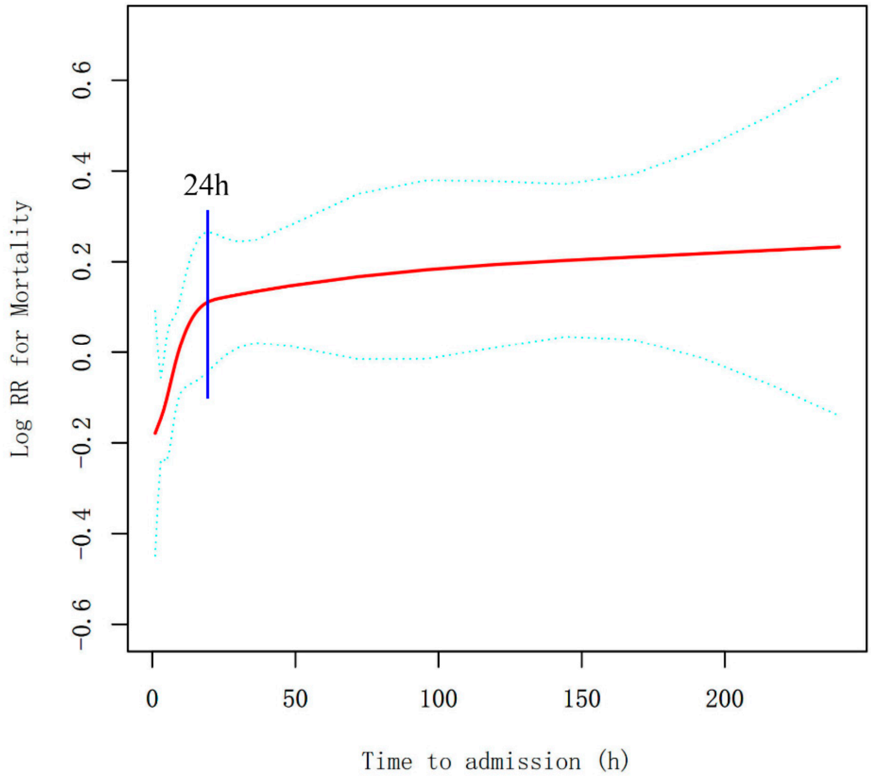

Supplementary Figure S1: A Cox model with restricted cubic spline plot illustrating the relationship between TTA and the risk of long-term mortality. The two-piecewise Cox regression analysis found the inflection point of 24h. When TTA was less than 24 hours, every one-hour increase in TTA was associated with a 1.6% increase in long-term mortality (HR=1.016, 95% CI: 1.008-1.024; *P*<0.001). When TTA exceeded 24 hours, the long-term mortality rate of patients became relatively stable and no longer changed with TTA (HR=1.000, 95% CI: 1.000-1.000; *P*=0.531).

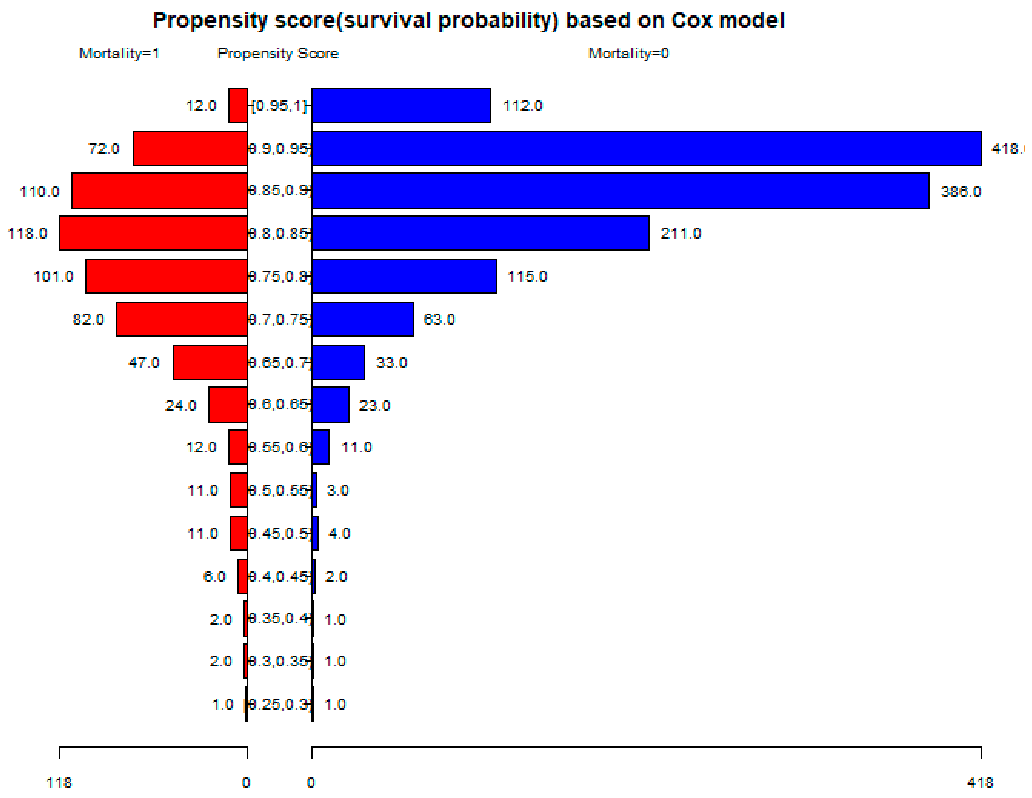

Supplementary Figure S2. The distribution of propensity scores after PSM.
